# The roles of the human SETMAR (Metnase) protein in illegitimate DNA recombination and non-homologous end joining repair

**DOI:** 10.1016/j.dnarep.2019.06.006

**Published:** 2019-08

**Authors:** Michael Tellier, Ronald Chalmers

**Affiliations:** School of Life Sciences, University of Nottingham, Queen’s Medical Centre, Nottingham, NG7 2UH, UK

**Keywords:** DSB, DNA double-strand break, NHEJ, non-homologous end joining, SETMAR, DNA integration, Non-homologous end joining, DNA double-strand break repair, Metnase

## Abstract

*SETMAR* is a fusion between a SET-domain methyltransferase gene and a mariner-family transposase gene, which is specific to anthropoid primates. However, the ancestral SET gene is present in all other mammals and birds. SETMAR is reported to be involved in transcriptional regulation and a diverse set of reactions related to DNA repair. Since the transcriptional effects of SETMAR depend on site-specific DNA binding, and are perturbed by inactivating the methyltransferase, we wondered whether we could differentiate the effects of the SET and MAR domains in DNA repair assays. We therefore generated several stable U2OS cell lines expressing either wild type SETMAR or truncation or point mutant variants. We tested these cell lines with *in vivo* plasmid-based assays to determine the relevance of the different domains and activities of SETMAR in DNA repair. Contrary to previous reports, we found that wild type SETMAR had little to no effect on the rate of cell division, DNA integration into the genome or non-homologous end joining. Also contrary to previous reports, we failed to detect any effect of a strong active-site mutation that should have knocked out the putative nuclease activity of SETMAR.

## Highlights

• Full length SETMAR expression has no effect on DNA repair and integration *in vivo.*
• SETMAR putative nuclease activity is not required *in vivo.*
• Separate expression of the SET and MAR domains affects DNA repair and integration.
• SETMAR isoform with a truncated SET-domain is specific to species containing the MAR domain.

## 1 Introduction

SETMAR is an anthropoid primate-specific fusion between a histone methyltransferase, which dimethylates histone H3 lysine 36 (H3K36me2), and a domesticated Hsmar1 transposase [[1], [2], [3]]. The transposase domain is 94% identical to the consensus sequence of the Hsmar1 transposase consensus sequence but three mutations, including the DDD to DDN mutation of the catalytic triad, completely abolish the transposition activity of SETMAR [[4], [5], [6]]. Although some activity, particularly 5′-end nicking, is recovered *in vitro* in the presence of DMSO and Mn^2+^, it is unlikely to be significant under physiological conditions [4]. Nevertheless, the transposase domain of SETMAR retains its ability to dimerize and bind transposon ends [2,4,7]. Recently, it was shown that the expression of thousands of genes is significantly altered in stable human cell lines when they overexpress a modest amount of SETMAR [7]. Furthermore, the pattern of transcriptional-changes induced by SETMAR expression depended on the methyltransferase and its site specific DNA binding to the Hsmar1 transposon ends scattered throughout the human genome [7]. This supports the hypothesis that the DNA binding domain of SETMAR serves to target the methylase to a subset of the Hsmar1 transposon ends dispersed throughout the human genome [2].

Previous SETMAR overexpression and knockdown experiments suggested that SETMAR was involved in illegitimate DNA integration and DNA repair through the non-homologous end joining repair (NHEJ) pathway [1]. NHEJ is one of the four pathways used by the cell to repair DNA double-strand breaks (DSBs) and the primary repair pathway throughout the cell cycle [8]. NHEJ is a template-independent DNA repair mechanism, which relies on Ku proteins to bind the DNA free ends, on nucleases, such as Artemis, or polymerases to trim or fill the DNA overhangs, and on the DNA ligase IV complex to ligate together the two blunt ends [8].

Although illegitimate DNA integration of transfected plasmid DNA involves the NHEJ pathway, the precise mechanism remains uncertain [9,10]. The current model states that the circular plasmid has to be linearized by a DSB to recruit DNA repair proteins on the plasmid ends [10]. For genomic integration to happen, one plasmid end needs to be in the vicinity of a genomic lesion for the NHEJ proteins to use the linearized plasmid DNA to repair the genomic DSB [10].

One of the difficulties in understanding the functions of SETMAR in DNA repair is that its overexpression produced a response in a number of different assays, suggesting that it was involved in many different aspects of DNA metabolism. For example, its overexpression promoted classical NHEJ, the random integration of transfected plasmid DNA and the restart of stalled replication forks [1,11]. Based on *in vitro* analysis, it has been hypothesized that purified SETMAR could act as an endonuclease like Artemis [12,13]. However, SETMAR endonuclease activity has only been established *in vitro* and recent reports question its relevance *in vivo* [13,14]. In contrast to Artemis, which promotes both trimming of DNA overhangs and DNA repair in cell extract assays, SETMAR did not stimulate DNA repair and only promoted trimming in one assay.

The SET methylase-domain of SETMAR was shown to interact with PRPF19, also known as PSO4, which is a protein involved in classical NHEJ and the spliceosome [15,16]. The interaction with PRPF19 was predicted to target SETMAR to double strand DNA breaks where the SET domain could dimethylate the histone H3 lysine 36 of neighbouring nucleosomes [17]. This epigenetic mark would recruit and stabilize Ku70 and NBS1 to the DNA ends [17]. Two other papers linked the increase in H3K36me2 following DSBs to the inhibition of KDM2A and KDM2B, two histone demethylases involved in the removal of H3K36 methylation [18,19]. However, a recent study using a genome-wide approach did not detect an increase in H3K36me2 around DSB sites [20].

A peculiarity of SETMAR is that it is a dimer in solution whereas almost all mammalian histone methyltransferases function as monomers [21,22]. The only known exception is vSET, a viral histone methyltransferase, which is active only as a dimer [23]. The crystal structure of the SET domain is also a monomer strengthening the hypothesis that the pre-fusion SET gene was operating as a monomer [24]. The MAR domain enforces the dimerization of SETMAR so even though the SET domain does not dimerize *sensu stricto*, the proximity between the two SET domains could however affect the methyltransferase activity or the interactions.

**Fig. 1:**
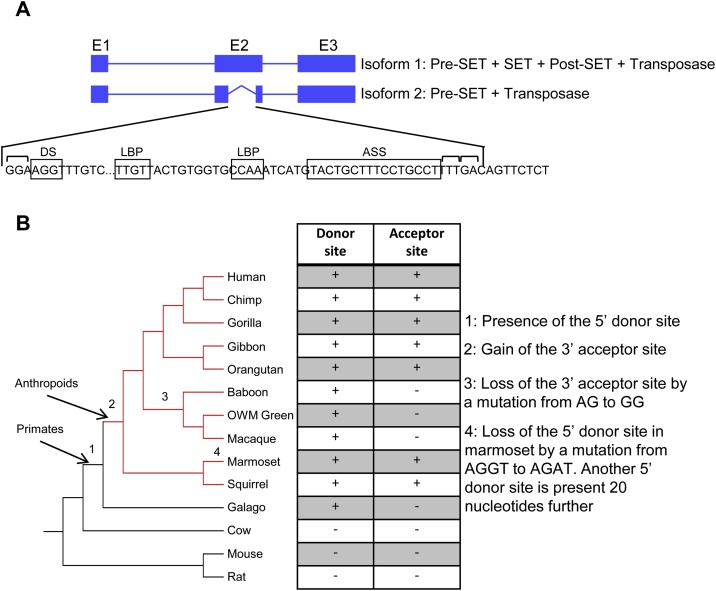
SETMAR second isoform is specific to anthropoid primates. **A**, The human *SETMAR* gene encodes two major isoforms, the full-length protein (isoform 1) and a truncated protein (isoform 2). The second isoform is methyltransferase deficient because the majority of the SET and post-SET domains are removed. Canonical donor site (DS), lariat branch points (LBP), and acceptor splicing site (ASS) are present in the second exon of SETMAR. The top brackets represents the exon codons. **B**, Phylogenetic tree of the second exon of SETMAR in several mammals. Although the 5′ donor site is absent in the marmoset [4], it must have appeared before the emergence of primates because it is present in several non-primates mammals. The 3′ acceptor site is specific to anthropoid primates except for the old-world monkeys which lost it owing to a single point mutation.

Interestingly, one isoform of SETMAR must have a defective methyltransferase activity because splicing removes the majority of the SET and post-SET domains in the second exon. This isoform is specific to the species where the SET gene is fused to the Hsmar1 transposase (Fig. 1). The 5′ donor site is present in primates and several other mammals but the acceptor site is only found in anthropoid primates, except for the old-world monkeys where a single mutation in their common ancestor abolishes the acceptor site. The marmosets also lost the 5′ donor site but another less conserved site is present 20 nucleotides away. This internally deleted isoform is expressed in most human tissues but at a lower level than the main isoform encoding the active methyltransferase [25]. This indicates that some SETMAR dimers would be composed of only one active SET domain and could function differently from SETMAR dimers with two SET domains.

To dissect the roles of SETMAR in illegitimate DNA integration and repair we created several U2OS cell lines expressing isolated SET and MAR domains, together with various point mutants to differentiate the effects of the methyltransferase, DNA binding and the putative nuclease activity. We found that stable over-expression of SETMAR did not significantly alter DNA integration, repair or the rate of cellular proliferation. The most significant effect was from overexpression of the SET domain, which increased plasmid integration and decreased the rate of plasmid end joining. This raises the possibility that the dimerization of SETMAR imposed by the MAR transposase domain might block the activities we observe for the isolated SET domain.

## 2 Materials and methods

### 2.1 Media and growth conditions

The T-Rex-U2OS cell lines were maintained in complete Dulbecco’s modified Eagle’s medium (DMEM, Sigma) supplemented with 10% heat inactivated Foetal Bovine Serum (FBS), 100 μg/ml of streptomycin, 100 μg/ml of penicillin, and 5 μg/ml of blasticidin (Sigma) at 37 °C with 5% CO_2_. The medium of T-Rex-U2OS cell lines stably expressing a gene of interest from an integrated pcDNA4TO plasmid was supplemented with 400 μg/ml of zeocin (InvivoGen).

### 2.2 Plasmids

An artificial codon-optimized version of SETMAR was synthesized by Gene Art (Thermo Fischer) and cloned into pcDNA4TO at the EcoRI/NotI restriction sites to produce pRC1702 (SM). Three Flag tags were cloned into pcDNA4TO between EcoRI and XbaI to produce pRC1703. The artificial codon-optimized version of SETMAR was cloned into pRC1703 at the HindIII/EcoRI restriction sites to produce pRC1704 (SMF). The truncated versions of SETMAR were produced by PCR and cloned into pRC1703 at the HindIII/EcoRI restriction sites to construct SETF (pRC1705) and MARF (pRC1706). SETF-N210A (pRC1707) and SMF-N210A (pRC1708), -R432A (pRC1709) and -D483A (pRC1710) single point mutant were produced by PCR on pRC1705 for SETF and pRC1704 for SMF. For the plasmid integration assay, pRC1714 was constructed by cloning a neomycin resistance gene into pBluescript SKII+ (Agilent) at the BamHI restriction site. Plasmid sequences are given in Supplemental Table 1.

**Table 1:** Mammalian cell lines used in this study.

Cell line	Description
T-Rex-U2OS	Human osteosarcoma cell line stably expressing the tetracycline repressor protein.
T-Rex-U2OS-TO (U2OS)	Parental line. T-Rex-U2OS cell line stably transfected with an empty pcDNA4TO.
T-Rex-U2OS-SETMAR	T-Rex-U2OS cell line stably expressing SETMAR.
T-Rex-U2OS-TO-Flag (U2OS-F)	Parental line. T-Rex-U2OS cell line stably transfected with an empty pcDNA4TO-Flag.
T-Rex-U2OS-SET-Flag	T-Rex-U2OS cell line stably expressing the Flag-tagged exons 1 and 2 of SETMAR (= SET domain).
T-Rex-U2OS-SET-Flag N210A	T-Rex-U2OS cell line stably expressing the Flag-tagged exons 1 and 2 of SETMAR (= SET domain) with the mutation N210A abolishing the methyltransferase activity of SET.
T-Rex-U2OS-MAR-Flag	T-Rex-U2OS cell line stably expressing the Flag-tagged exon 3 of SETMAR (= MAR domain).
T-Rex-U2OS-SETMAR-Flag	T-Rex-U2OS cell line stably expressing a Flag-tagged SETMAR.
T-Rex-U2OS-SETMAR N210A-Flag	T-Rex-U2OS cell line stably expressing a Flag-tagged SETMAR with the mutation N210A abolishing the methyltransferase activity of SETMAR.
T-Rex-U2OS-SETMAR R432A-Flag	T-Rex-U2OS cell line stably expressing a Flag-tagged SETMAR with the mutation R432A decreasing the affinity of SETMAR for the transposon end.
T-Rex-U2OS-SETMAR D483A-Flag	T-Rex-U2OS cell line stably expressing a Flag-tagged SETMAR with the D483A mutation abolishing the catalytic activity of transposase domain of SETMAR.

### 2.3 Stable transfection of T-Rex-U2OS cells

For each transfection, 2.5 × 10^5^ cells were seeded in a 6-well plate and grown overnight in DMEM supplemented with 10% FBS. The plasmids were transfected using Lipofectamine 2000 (Invitrogen), following manufacturer’s instruction. After 24 h, a quarter of the cells were transferred to 100 mm dishes and the medium supplemented with 400 μg/ml of zeocin (InvivoGen). After 2 weeks of selection, single foci were picked and grown in a 24-well plate. The expression of the gene of interest was verified in each cell line by inducing the PCMV promoter with doxycycline at a final concentration of 1 μg/ml for 24 h. The list of cell lines used in this study is presented in Table 1.

### 2.4 Western blotting

Whole cell extracts were harvested from cultures at ˜90% confluency in six-well plates. Briefly, cells were washed twice with ice-cold PBS then pelleted for 5 min at 3000 x g at 4 °C. Samples were resuspended in 100 μl of Radio ImmunoPrecipitation Assay (RIPA) buffer (10 mM Tris−HCl pH8.0, 150 mM NaCl, 1 mM EDTA, 0.1% SDS, 1% Triton X-100, 0.1% sodium deoxycholate) with freshly added protease inhibitor cocktail (Roche Applied Science) and incubated on ice for 30 min with a vortexing every 10 min. Cell lysates were centrifuged for 15 min at 14,000 x g at 4 °C and the protein in the supernatants was quantified by the Bradford assay.

For each western blot, 20 μg of cell-extract protein was mixed with 2X SDS loading buffer, boiled for 5 min, and electrophoresed on a 10% SDS-PAGE gel. Proteins were transferred to a polyvinylidene difluoride (PVDF) membrane, which was blocked in 5% milk or BSA (Roche) and incubated with specific primary antibodies at 4 °C overnight. After washing, membranes were incubated with horseradish peroxidase (HRP)-conjugated secondary antibodies for one hour at room temperature, washed, and signals were detected with the ECL system (Promega) and Fuji medical X-ray film (Fujifilm).

The following antibodies were used: anti-beta Tubulin (rabbit polyclonal IgG, 1:500 dilution, ab6046, Abcam), anti-Hsmar1 antibody (goat polyclonal, 1:500 dilution, ab3823, Abcam), anti-Flag (rabbit, 1:500 dilution, F7425, Sigma). The secondary antibodies were horseradish peroxidase-conjugated anti-goat (rabbit polyclonal, 1:5000 dilution, ab6741, Abcam) and anti-rabbit (goat polyclonal, 1:5000-1:10000, ab6721, Abcam).

### 2.5 Transcriptome acquisition and analysis

Total RNAs were isolated from cells grown to ∼80% confluency in six-well plate with the High Pure RNA isolation kit (Roche Applied Science), following manufacturer's instructions. The samples were quantified with a Nanodrop Spectrophotometer and their quality verified with a Bioanalyzer (Agilent). Only samples with a RIN number >9 were used. Approximately 10 μg of total RNA was used to enrich for mRNA using two rounds of enrichment with Dynabeads Oligo(dT)25 (Life tech, 61,005). Solid whole transcriptome libraries were made according to the Solid Total RNASeq protocol (Life tech, 4,445,374) and sequenced on an ABi SOLiD 5500xl analyzer according to the manufacturer's instructions to generate 50 bp reads in colour space. RNA-seq was performed on biological duplicates. The RNA-seq colour-space reads were initially processed using The LifeTechnologies LifeScope (v2.5.1) Whole Transcriptome Pipeline. Reads were first filtered against rRNA, tRNA, and sequencing contaminants and then mapped to the Homo sapiens GRCh37 hg19 genome assembly from Ensembl. For calculating the reads per kilobase of transcript per million mapped reads (RPKM) values, exons were extracted in R with the GenomicFeatures package and grouped by ‘gene’. Expression at the transcript level was quantified with HTSeq version 0.6.1 and differential expression was calculated with the DESeq2 software version 3.2, keeping only the genes with a fold change <−2 or >2 and an adjusted P-value of 0.05. Sequencing data have been deposited in GEO under accession number GSE129870.

### 2.6 Growth rate

On day 0, 2 × 10^4^ cells were seeded in eight 6 cm dishes for each cell line and one dish was counted every day for eight days using a hemocytometer. A p-value less than or equal to 0.01 was considered as statistically significant.

### 2.7 Illegitimate DNA integration assay

For integration assays in the Flag-tagged T-Rex-U2OS cell lines, 8 × 10^5^ cells were seeded onto 6-well plates with 2.5 μg of circular or linearized pRC1712 and 5 μl of Lipofectamine 2000 (Invitrogen). Twenty-four hours later, cells were trypsinized and 5 × 10^4^ cells of each transfection were seeded onto 10 cm dishes in medium containing 800 μg/ml of G418 (Sigma). For integration assays in U2OS and SM3, 10^5^ cells were seeded onto 6-well plates with 2 μg of circular pRC1712 and 4 μl of Lipofectamine 2000 (Invitrogen). Twenty-four hours later, cells were trypsinized and the cells of each transfection were seeded onto 10 cm dishes in medium containing 800 μg/ml of G418 (Sigma). After two weeks of selection, surviving foci were fixed for 15 min with 10% formaldehyde in PBS, stained for 30 min with methylene blue buffer (1% methylene blue, 70% ethanol), washed with water, air dried, and photographed. The transfection efficiency was tested by transfecting a pEGFP plasmid. After 24 h, the live cells were observed using a Carl Zeiss Axiovert S100 TV Inverted Microscope with an HBO 100 illuminator. The transfection efficiency was found to be similar between the different cell lines. A p-value less than or equal to 0.01 was considered as statistically significant.

### 2.8 Non-homologous end-joining assay and FACS analyses

Prior to transfection, the pEGFP-Pem1-Ad2 plasmid was digested overnight with HindIII or I-SceI. The digestion reactions were heat-inactivated and column-purified before being co-transfected with a pRFP plasmid as a control for transfection efficiency. A day before transfection, 8 × 10^5^ cells were seeded in 60 mm dishes to obtain a ˜70% confluency on the transfection day. Transfections were performed with 3 μg of linear pEGFP-Pem1-Ad2, 3 μg of pRFP and 14 μl of Lipofectamine 2000 (Invitrogen), according to manufacturer’s instructions. After 24 h, green (GFP) and red (RFP) fluorescence was measured by fluorescence-activated flow cytometry (FACS). For FACS analysis, cells were harvested with Accutase (Sigma), washed once in 1X PBS and fixed in 2% formaldehyde (Sigma). FACS analysis was performed on a Coulter FC500 (Beckman Coulter) and data analysed using Weasel software v3.0.2. The numbers of repaired events are reported as the ratio of green and red positive cells over the total number of red positive cells. This ratio normalizes the numbers of repaired events to the transfection efficiency. The values for all the cell lines are reported as a percent of the control cell lines. A p-value less than or equal to 0.01 was considered as statistically significant.

## 3 Results

### 3.1 SETMAR overexpression does not promote U2OS cell proliferation

**Fig. 2:**
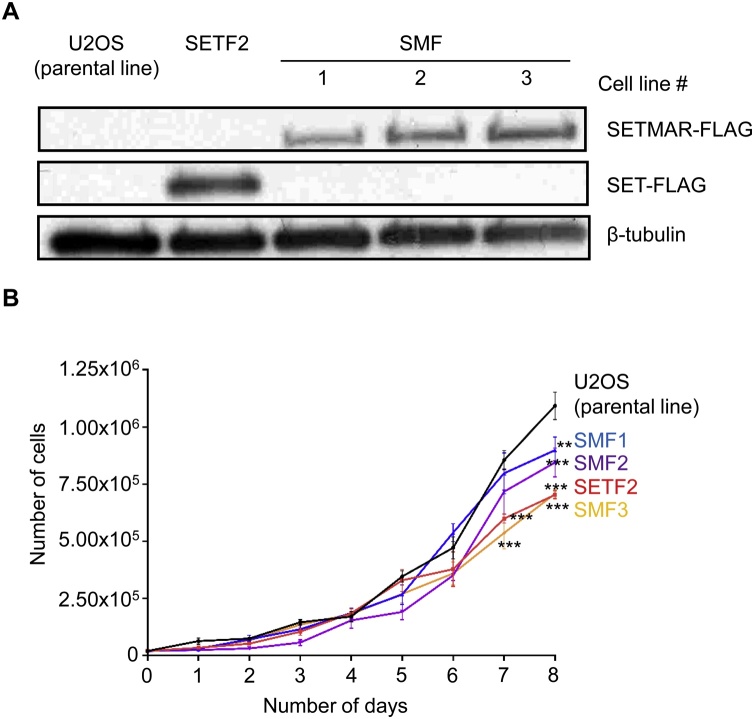
SETMAR overexpression does not promote U2OS cell proliferation. **A**, Western blot for the Flag-tagged SETMAR in the U2OS, SETF and SMF cell lines. The western blot was performed with anti-Flag and anti-β-tubulin antibodies. **B**, The growth rate of U2OS, SETF and SMF cell lines. On day 0, 2.0 × 10^4^ cells were seeded in eight dishes and one dish was counted every day for eight days. Average ± S.E.M. of 3–5 biological replicates. Statistical test: *t*-test with Holm-Sidak correction, ** p-value < 0.01, *** p –value < 0.001.

It was previously reported that SETMAR overexpression increased the growth rate of the HEK293 and HEK293 T cell lines [26]. Conversely, SETMAR depletion by RNA interference or CRISPR/Cas9 knock-out decreased the growth rate of THP-1 and DLD-1 cancer cells, respectively [27,28]. Furthermore, we previously demonstrated that modest, stable, overexpression of SETMAR in a U2OS cell line significantly changed the expression of a large set of genes, and that this depended on the DNA binding and methyltransferase activities [7]. However, we did not detect an enrichment for genes involved in the cell cycle [7]. To determine whether altering SETMAR expression level also affects the growth rate of the U2OS cell line, we tested three stable T-Rex-U2OS cell lines overexpressing SETMAR at different levels, together with one cell line expressing the SET domain only and a parental cell line with an empty expression vector integrated in its genome (Fig. 2A). The expression level of the SET domain or SETMAR was determined by western blotting using an anti-Flag antibody. The growth rate was determined by counting the number of cells across a period of eight days (Fig. 2B). Contrary to previous reports, we observed a small but significant decrease in cell proliferation for most of the cell lines overexpressing SET or SETMAR after 7 days.

### 3.2 Effects of expressing SETMAR mutants and individual domains

**Fig. 3:**
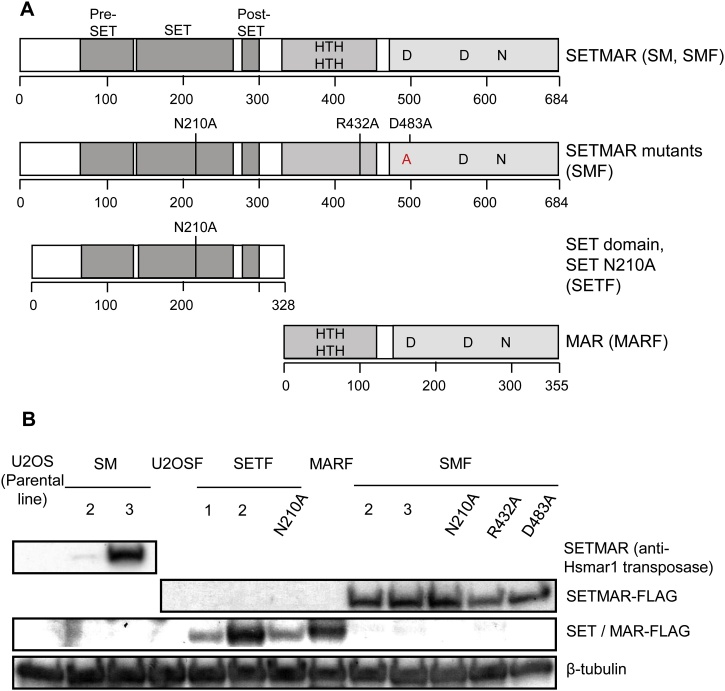
U2OS cell lines used in the *in vivo* DNA repair assay. **A**, A schematic representation of SETMAR (pRC1702 and pRC1704), SET (pRC1705) and MAR (pRC1706) and the locations of the different mutations (pRC1707 to pRC1710). **B**, A western blot for the Flag-tagged SETMAR in the U2OS, SM, SETF, MARF and SMF cell lines. The western blot was performed with anti-Hsmar1 transposase (SETMAR), anti-Flag (SETMAR-Flag, SET / MAR-Flag) and anti-β-tubulin antibodies. The cell lines are described in Table 2. U2OS, U2OSF: parental lines; SM: SETMAR; SMF: SETMAR-Flag; SETF: SET domain-Flag; MARF: MAR domain-Flag.

To improve our understanding of the roles of SETMAR in illegitimate DNA integration and the NHEJ pathway, we produced several U2OS cell lines stably overexpressing wild type, truncated or mutant versions of SETMAR (Fig. 3A and Table 2). Previously, we found that transient transfection of a SETMAR expression vector gave 2500-fold overexpression [7]. We therefore established stable cell lines to obtain more modest expression levels. We relied on an overexpression strategy rather than knockdown because the affinity of the commercial antibodies was too low to detect expression of endogenous SETMAR in the U2OS cell line. To interpret the experiments we must recall that the purified transposase domain exists as a dimer and that this is probably also the multimeric state of SETMAR *in vivo* [29]. The concentration of endogenous SETMAR in the U2OS cell line is relatively low, with less than 500 molecules per cell [30]. Since the exogenous proteins were expressed to a much higher level, most of the multimers will be homogeneous. However, the endogenous SETMAR will produce a low level of mixed multimers by random assortment. It is therefore important to keep in mind that the activity of the endogenous SETMAR may be influenced by the overexpression of the mutant subunits. The exogenous expression level of each cell line was determined by western blotting using anti-Hsmar1 transposase and anti-Flag antibodies (Fig. 3B). An anti-Flag antibody was used for the cell lines containing an F (for Flag-tag) in their names. SM2 and 3 cell lines overexpress untagged versions of SETMAR so their expression levels were determined using an antibody against the last nine amino acids of SETMAR.

**Table 2:** U2OS cell lines used in the *in vivo* DNA repair assay.

Full name	Abbreviation	Expression level	Comparison to SMF3 cell line on western blots
T-Rex-U2OS-TO (parental line)	U2OS	Endogenous level	/
T-Rex-U2OS-SETMAR	SM2	High	Visible with anti-Hsmar1 transposase antibody, not SMF3
	SM3	Very high	Visible with anti-Hsmar1 transposase antibody, not SMF3
T-Rex-U2OS-TO-Flag (parental line)	U2OS-F	Endogenous level	/
T-Rex-U2OS-SET-Flag	SETF1	Low	Lower than SMF3 with anti-Flag antibody
	SETF2	Medium/High	Higher than SMF3 with anti-Flag antibody
T-Rex-U2OS-SET-N210A- Flag	SETF-N210A	Low	Lower than SMF3 with anti-Flag antibody
T-Rex-U2OS-MAR-Flag	MARF	Medium/High	Higher than SMF3 with anti-Flag antibody
T-Rex-U2OS-SETMAR-Flag	SMF1	Medium	Similar to SMF3 with anti-Flag antibody
	SMF2	Medium	Similar to SMF3 with anti-Flag antibody
	SMF3	Medium	/
T-Rex-U2OS-SETMAR-N210A-Flag	SMF-N210A	Medium	Similar to SMF3 with anti-Flag antibody
T-Rex-U2OS-SETMAR R432A-Flag	SMF-R432A	Low	Lower than SMF3 with anti-Flag antibody
T-Rex-U2OS-SETMAR D483A-Flag	SMF-D483A	Low/Medium	Lower than SMF3 with anti-Flag antibody

The two control cell lines, U2OS and U2OS-F, express only the endogenous SETMAR. We used four cell lines overexpressing wild type SETMAR at medium (SMF2 and SMF3), high (SM2) or very high levels (SM3). The relative levels of SETMAR expression in SMF3, SM2, and SM3 were first confirmed by western blotting with the anti-Hsmar1 transposase antibody (Supplementary Fig. 1). A signal was observed only for SM2 and SM3, indicating that SMF3 expresses SETMAR at a lower level than SM2 and SM3. We also performed RNA-seq on the U2OS, SM2, and SM3 cell lines and compared the expression levels to the RNA-seq experiments that we previously conducted in the SMF3 cell line (Table 3) [7]. Since we used two different RNA-seq methods (SOLiD for the SM cell lines and Illumina for the SMF3 cell line), we normalized the ectopic SETMAR expression to β-tubulin. Consistent with the result of the western blot, we found the normalized SETMAR expression to be three times lower in SMF3 than in SM2. Of note, in the SM3 cell line exogenous SETMAR is the 32^nd^ most expressed gene, compared to the endogenous SETMAR which is the 8,377^th^ most expressed gene.

**Table 3:** SETMAR and β-tubulin expression levels in the U2OS cell lines expressing full-length SETMAR.

Cell line	*SETMAR* (RPKM/FPKM)	*TUBB* (RPKM/FPKM)	% of *TUBB*
U2OS	4	336	1
SM2	193	283	68
SM3	410	248	166
U2OS-F	5	976	1
SMF3	176	818	22

In the SETF1 and SETF2 cell lines, the SET domain is expressed at low and medium-to-high levels, respectively. In the MAR cell line, the transposase domain is expressed at medium-to-high level. We also created three point mutations in key functional residues. The N210A mutation, which is located in the key NHSC motif of the SET domain, abolishes the methyltransferase activity of SETMAR [7]. To investigate any effect of SETMAR binding to genomic Hsmar1 transposon ends (inverted terminal repeat, ITR), we introduced the R432A mutation, which decreases the affinity of SETMAR for the Hsmar1 transposon ends [29,31]. To test the requirement of SETMAR’s putative nuclease activity, we introduced the D483A mutation. This substitutes the first D in the DDD triad of amino acids that coordinate the catalytic metal ions in the active site [13,29]. Although some substitutions of the last D residue may be tolerated, the first D coordinates both metal ions in the active site and is absolutely required for activity in the RNase H family of nucleases [32].

### 3.3 The SET and MAR domains but not SETMAR promote DNA integration

**Fig. 4:**
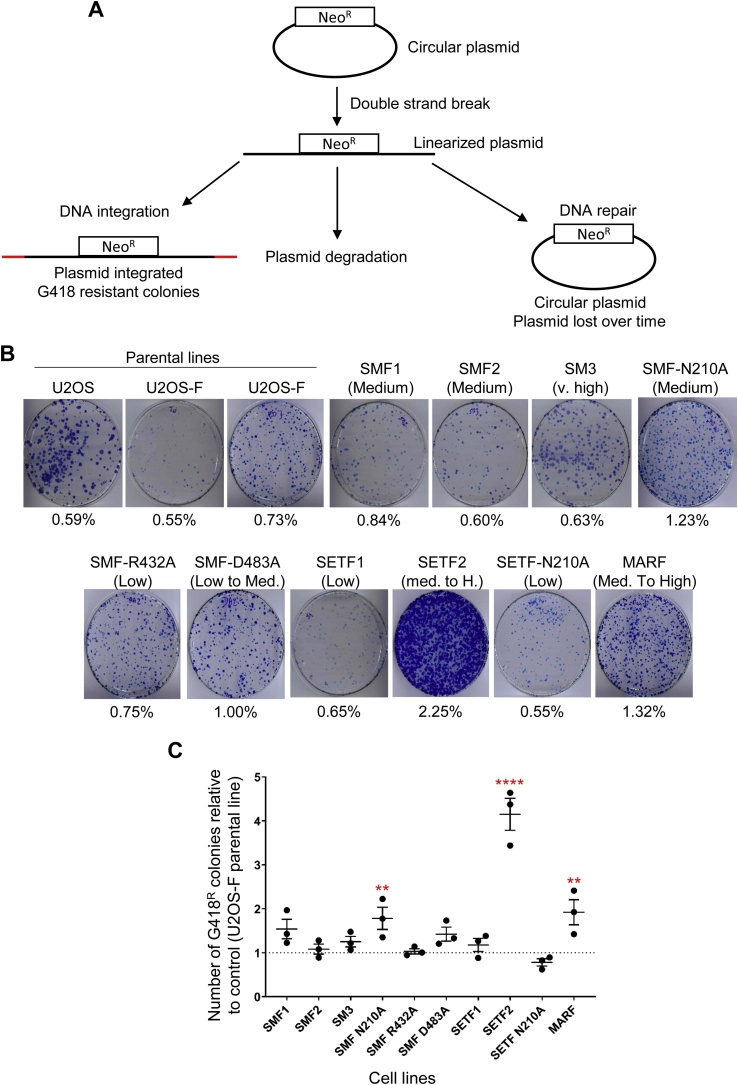
The SET and MAR domains but not SETMAR promote DNA integration. **A**, A schematic representation of the integration assay. Cells are transfected with a circular plasmid (pRC1714), which encodes a neomycin resistance gene. For integration to occur through the NHEJ pathway, the plasmid must be linearized by a DSB and a plasmid free end has to be in close vicinity of a genomic DSB. The linearized plasmid can also be re-circularize or be degraded. Following G418 treatment for two weeks, cells in which the G418 marker has been integrated into the genome form foci which can be detected by methylene blue staining. **B**, Representative pictures of integration plates. The expression level is indicated between brackets and is derived from Table 2, Table 3. The integration rate for each cell line is indicated below each picture. **C,** DNA integration efficiency of a circular plasmid encoding a neomycin resistance gene in the different cell lines relative to the parental cell line, U2OS or U2OS-F. Average ± S.E.M. of 3 biological replicates. Statistical test: one-way ANOVA Dunnett’s multiple comparisons test, ** p-value < 0.01, **** p-value < 0.0001.

SETMAR was previously reported to promote the illegitimate integration of plasmid DNA into the genome [1]. We used the different Flag-tagged SETMAR domains and mutants to gain a better understanding of DNA integration. For a plasmid to be integrated, two events are thought to be required (Fig. 4A). First, the plasmid must be linearized by a DSB and then it must be recruited by NHEJ proteins that are in the process of dealing with a chromosomal double strand break [10]. However, illegitimate integration is only one of the three possible outcomes for a linearized plasmid because it can also be re-circularized or degraded (Fig. 4A). We determined the rate of illegitimate plasmid integration by transfecting a plasmid encoding a neomycin resistance gene before challenging the cells with G418 for two weeks. Cells in which the plasmid has been integrated into the genome could develop into foci. The foci were counted after staining with methylene blue.

To determine whether the topology of the plasmid influences its integration frequency, we transfected a circular or linear version of the same plasmid in the control cell line U2OS-F. We observed a ˜3-fold decrease in the integration of the linearized form compared to the circular (Supplementary Fig. 2). We therefore used the circular plasmid for all further experiments with SETMAR and its derivatives.

We measured the rate of plasmid integration with cell lines expressing SETMAR and a selection of mutant derivatives including its individual domains (Fig. 4B and C). Since the respective genes are integrated in the genome, the protein expression levels are not identical and a strict comparison between the cell lines is not always possible (Table 2,3). Nevertheless, since the cell lines are clonal, and each cell in the respective population has the same expression level, this strategy is better than transient transfection where there is a distribution of expression-cassette copy-numbers. In three cell lines expressing wild type SETMAR at medium to very high levels we detected no significant difference in the plasmid integration rate (Fig. 4B and C). There were marginal increases in integration rate with the MAR domain and the SETMAR N210A mutant. The largest and most significant increase was for the cell line expressing a medium to high level of the SET domain.

### 3.4 The SET and MAR domains have an opposite effect on plasmid end-joining

**Fig. 5:**
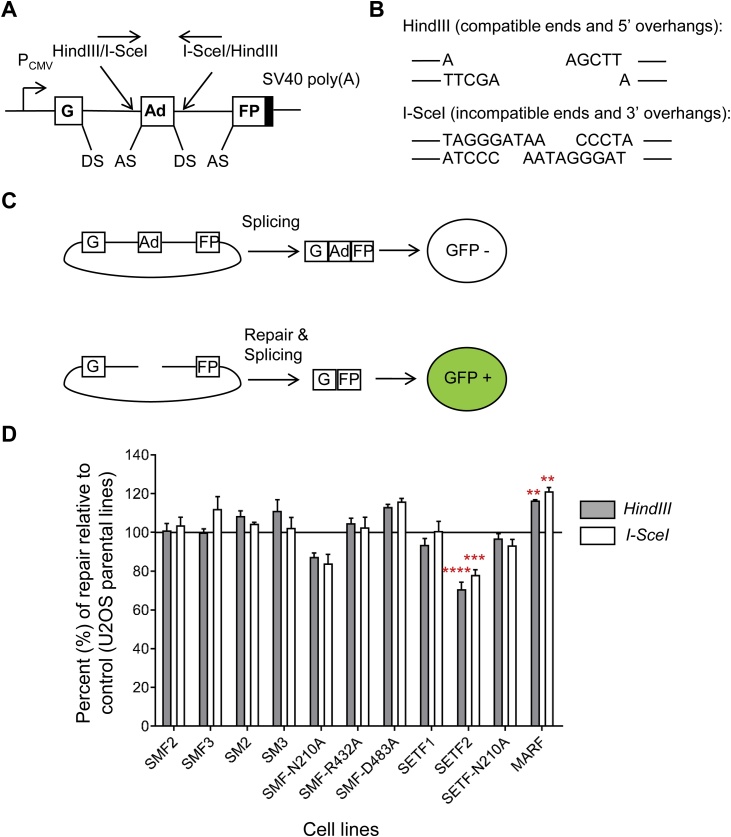
The SET and MAR domains have an opposite effect on DNA repair. **A**, The reporter plasmid, pEGFP-Pem1-Ad2, is composed of a GFP cassette flanked by a PCMV promoter and a SV40 poly(A) sequence. The eGFP coding sequence is interrupted by a 2.4 kb intron containing an adenovirus exon (Ad). The Ad exon is flanked by *HindIII* and *I-SceI* restriction sites. The donor (DS) and acceptor (AS) splicing sites are shown. **B***, HindIII* and *I-SceI* restriction sites are respectively composed of a palindromic 6-bp and a non-palindromic 18-bp sequence. Digestion of the reporter by HindIII or I-SceI generates respectively compatible and incompatible ends. **C**, The presence of the Ad exon in the eGFP ORF inactivates the eGFP activity thus making the cell eGFP negative. Removal of the Ad exon by HindIII or I-SceI followed by a successful intracellular repair will restore eGFP expression, which is then quantified by flow cytometry. The assay was adapted from ref. [33]. **D**, The frequency of end joining in the indicated cell lines compared to the parental U2OS and U2OS-F cell lines was determined by flow cytometry. Average ± S.E.M. of 3 biological replicates. Statistical test: one-way ANOVA Dunnett’s multiple comparisons test, ** p-value < 0.01, *** p-value < 0.001, **** p-value < 0.0001.

To gain a better understanding of SETMAR functions in the NHEJ pathway, we used an established *in vivo* DNA repair assay [33]. The assay is based on two plasmids, one encoding the reporter gene (pEGFP) and the other serving as a transfection control (pRFP). The reporter plasmid encodes an *EGFP* gene interrupted by a 2.4 kb intron derived from the rat *Pem1* gene. An exon from the adenovirus (Ad2) has been integrated in the intron, which abolishes the GFP activity (Fig. 5A). The Ad2 exon is flanked by *HindIII* and *I-SceI* recognition sites. Cleavage with HindIII or I-SceI yields compatible or incompatible ends, respectively (Fig. 5B). These two types of ends require different steps for repair. Compatible ends can be ligated directly, while incompatible ends with 3′-overhangs have to be trimmed before ligation. The repair of the linearized plasmid by the NHEJ pathway restores the GFP ORF (Fig. 5C). The repair events were detected by flow cytometry measuring at least 10,000 cells per assay. The repair efficiency was calculated as the ratio of green and red cells over the total number of red cells, thus normalizing the transfection efficiency between cell lines.

To normalize between the biological replicates, we calculated for each replicate the ratio of the repair efficiency of each cell line relative to their respective control cell lines. The average ratio from three independent experiments is presented in Fig. 5D. The SM2 and 3 cell lines were compared to the U2OS control cell line, whereas the remaining cell lines were compared to U2OS-F to take into account any possible effect of the Flag tag. Unsurprisingly, there was no significant difference in the repair efficiency of both types of ends between the two control cell lines (Supplementary Fig. 3). In agreement with previously published results for this assay, we observe that in most cell lines incompatible ends were repaired about 10% less efficiently than compatible ends [33]. Presumably this reflects the necessity of processing the DNA overhangs before the plasmid ends can be ligated.

The overexpression of SETMAR, irrespective of the level, or of SETMAR mutants did not promote the repair of either compatible or incompatible ends (Fig. 5D). This contradicts previous reports of *in vivo* experiments [1,34] but confirms later results in cell-free extracts [13]. However, with medium-to-high expression of the SET domain we detected a decrease of about 20% in the repair of both compatible and incompatible ends. In contrast, medium overexpression of the MAR domain increased the repair efficiency of both types of ends by ˜20% compared to the control cell line.

## 4 Discussion

A set of previous reports has claimed a direct role for SETMAR in diverse aspects of DNA metabolism [1,11,12,[15], [16], [17],^26^,^27^,^29^,[34], [35], [36], [37], [38]]. However, subsequent studies have challenged some of these claims to various extents [4,5,7,13,14,28].

One proposition was that SETMAR could be involved in DNA repair through the NHEJ pathway. Here we have pursued this question by investigating the relative contributions to NHEJ of SETMAR, the isolated SET and MAR domains, and, by way of mutagenesis, the contributions of the methyltransferase, the ITR binding and the nuclease activities. We found that expression of the SET domain, and to a lesser extent the MAR domain, but not wild type SETMAR, have a mild effect on DNA integration and end-joining. In addition, we found that the putative nuclease activity of SETMAR does not seem to be important for these processes *in vivo*.

Previous reports have linked SETMAR over-expression or knock-down/knock-out to increased or decreased proliferation, respectively, in HEK293, THP1, and DLD-1 cell lines [[26], [27], [28]]. We did not detect this effect in the U2OS cell line (Fig. 1B). Indeed, a modest overexpression of SETMAR or the SET domain reduced the growth rate slightly after six to seven days. Furthermore, in a previous transcriptomic study, and in the current RNA-seq measurements performed on the SM2 and SM3 cell lines, we did not detect an enrichment in cell-cycle genes in the set of genes differentially expressed upon SETMAR overexpression (Supplementary Table 2) [7].

Other previous reports on the function of SETMAR in NHEJ claimed two specific roles for the SET and the MAR domains [17,30]. Firstly, they proposed that the SET domain dimethylates H3K36 of nucleosomes near DSBs. This epigenetic mark is thought to recruit and stabilize the binding of Ku70 and NBS1 to the DNA ends [17]. Secondly, they proposed that the MAR domain trims DNA overhangs before other NHEJ proteins mediated ligation of the ends [12]. It has also been claimed that SETMAR activity is regulated by several interactions with other proteins involved in the NHEJ such as PRPF19 and DNA ligase IV [15,16]. The only confirmed direct interaction was between the SET domain and PRPF19 and was hypothesized to promote the recruitment of SETMAR to DSBs [15]. Based on our transcriptomic experiments in U2OS cell lines expressing SETMAR or SETMAR N210A, we did not observe any enrichment for DNA repair genes in the set of differentially expressed genes, indicating that SETMAR is unlikely to act in NHEJ through regulation of gene expression (Supplementary Table 2) [7].

In our *in vivo* assays, overexpression of the wild type SETMAR did not affect DNA end-joining or integration. However, we found that medium overexpression of the SET domain decreases DNA end-joining efficiency and increases illegitimate DNA integration (Fig. 4B and 5D, compare SETF2 to SETF1 and the control). In our assays, both DNA end-joining and integration are supposed to be dependent on the NHEJ pathway, consistent with a role for the ancestral SET gene in this pathway. However, the mechanism by which the SET domain favours DNA integration over DNA end-joining is unclear. The decrease in re-circularization with both compatible and incompatible ends found in the DNA end-joining assay could delay the re-circularization of plasmids, increasing the window of opportunity for a plasmid end to be in the vicinity of a genomic end and therefore promoting its genomic integration.

The clearest result of our assays was that overexpression of the MAR domain stimulates both DNA repair and integration (Fig. 4B and 5D). Amongst the set of previous reports proposing a direct role for SETMAR in diverse aspects of DNA repair there were claims that the MAR domain could bind DNA ends and its nuclease activity could trim the overhangs [12,13]. This was at odds with our own work in which we failed to detect any nuclease activity of the purified MAR domain using an extremely sensitive assay with a ^32^P-labelled substrate [4]. We detected faint nuclease activity if the DDN triad in the active site was mutated back to the wild type DDD or if the reaction was supplemented with Mn^2+^ instead of Mg^2+^. Since Mn^2+^ is not present in significant quantities *in vivo* we were sceptical about a nucleolytic role for SETMAR. Furthermore, if the MAR domain has a trimming activity, we would expect to observe a larger increase in the repair of incompatible ends *versus* compatible ends in the present experiments. In fact, the increase in the DNA end-joining is similar for both types of ends, which does not support a trimming activity (Fig. 5D). Also, overexpression of SETMAR D483A mutant, which should abolish any remaining catalytic activity of the MAR domain, does not decrease DNA end-joining (Fig. 5D). In fact, we observe no significant change on DNA integration and DNA end-joining (Figs. 4B and 5 D). This seems to indicate that the MAR domain of SETMAR does not trim DNA overhangs *in vivo*.

Unsurprisingly, the ITR binding activity of SETMAR is not required for DNA end-joining and integration (Figs. 4B and 5 D). In contrast, a medium overexpression of the methyltransferase defective mutant, SETMAR N210A, does not affect DNA end-joining but increases DNA integration, whereas overexpression of the wild type SETMAR does not affect either of these processes (Figs. 4B and 5 D). The absence of an effect of the N210A mutation in the SET domain is likely due to its low level of expression. Its level of expression is similar to SETF1, which is too low to affect DNA end-joining and integration in our assays. However, it remains unclear whether this effect on NHEJ of SETMAR N210A is mediated by a direct methylation of factors involved in NHEJ or a consequence of a decreased bulk H3K36me2 level in the SETMAR N210A cells [7]. Two studies, which also observed an increase in H3K36me2 at DSB sites, linked the increase to the removal of histone demethylases from the chromatin rather than active methylation [18,19]. In contrast, a recent study did not find any increase in H3K36me2 at a DSB site but found instead an increase in H3K36me3 [20]. In addition to a decreased H3K36me2 level, we also observed a decrease of H3K36me3 at some genomic positions, possibly because of a decrease in the level of H3K36me2, which is required by SETD2 for adding the third methyl group [7]. The decreased NHEJ activity with SETMAR N210A could therefore be owing to a reduced cellular level of H3K36me2/me3, which could affect the efficiency of repair by the NHEJ pathway.

An interesting question is why the SET and the MAR domains have an effect on DNA end-joining and integration but not the wild type SETMAR? One possibility is that the functions of the SET domain in NHEJ are blocked by the dimerization of the protein mediated by the MAR domain.

## Author contributions

Performed the experiments: MT. Conceived and designed the experiments and analysed the data, MT, RC. Wrote the paper: MT, RC.

## Funding

This work was supported by the Biotechnology and Biological Sciences Research Council [grant number BB/J014508/1]. Funding for open access charge: BBSRC [BB/J014508/1].
