# Supplementary material for: The roles of the human SETMAR (Metnase) protein in illegitimate DNA recombination and non-homologous end joining repair
Source: DNA Repair (Amst). 2019 Aug;80:26–35. doi: 10.1016/j.dnarep.2019.06.006 (PMC6715855; doi:10.1016/j.dnarep.2019.06.006)
Supplement: Supplementary file 1 [file mmc1.pptx]

## Slide 1
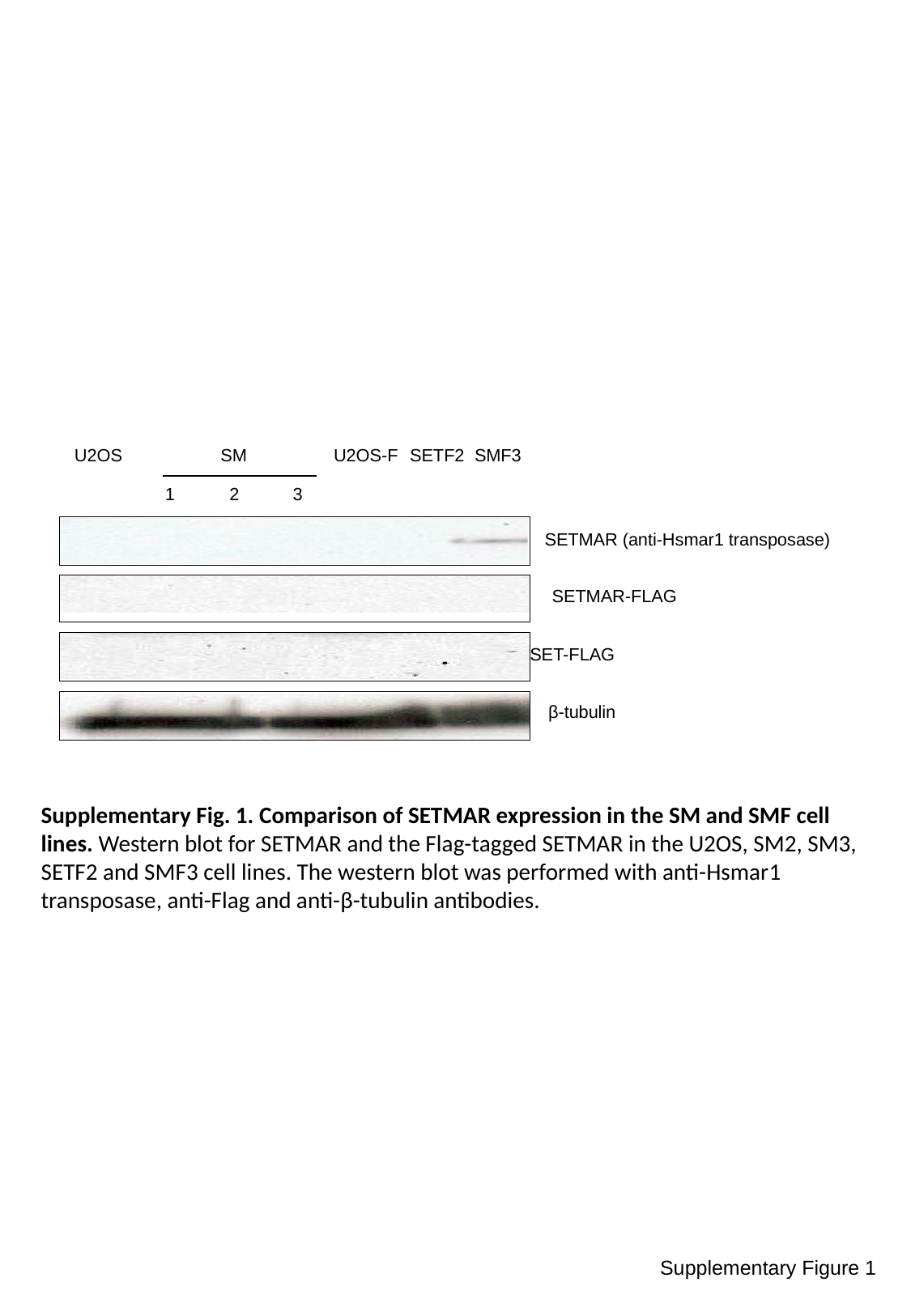

U2OS
SM
U2OS-F
SETF2
SMF3
1
2
3
SETMAR (anti-Hsmar1 transposase)
SETMAR-FLAG
SET-FLAG
β-tubulin
Supplementary Fig. 1. Comparison of SETMAR expression in the SM and SMF cell lines. Western blot for SETMAR and the Flag-tagged SETMAR in the U2OS, SM2, SM3, SETF2 and SMF3 cell lines. The western blot was performed with anti-Hsmar1 transposase, anti-Flag and anti-β-tubulin antibodies.
Supplementary Figure 1

## Slide 2
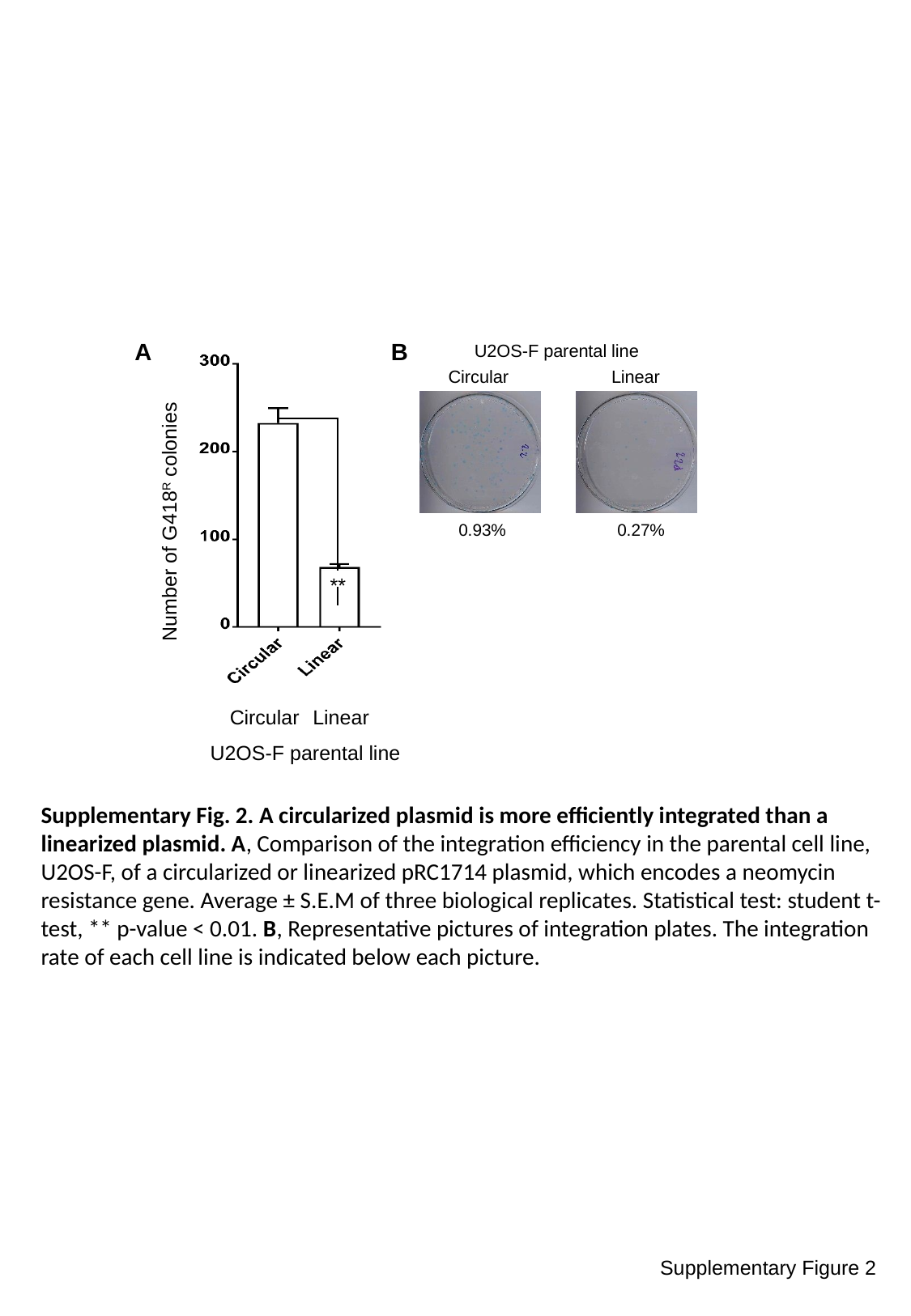

A
B
U2OS-F parental line
Circular
Linear
Number of G418R colonies
0.93%
0.27%
**
Circular
Linear
U2OS-F parental line
Supplementary Fig. 2. A circularized plasmid is more efficiently integrated than a linearized plasmid. A, Comparison of the integration efficiency in the parental cell line, U2OS-F, of a circularized or linearized pRC1714 plasmid, which encodes a neomycin resistance gene. Average ± S.E.M of three biological replicates. Statistical test: student t-test, ** p-value < 0.01. B, Representative pictures of integration plates. The integration rate of each cell line is indicated below each picture.
Supplementary Figure 2

## Slide 3
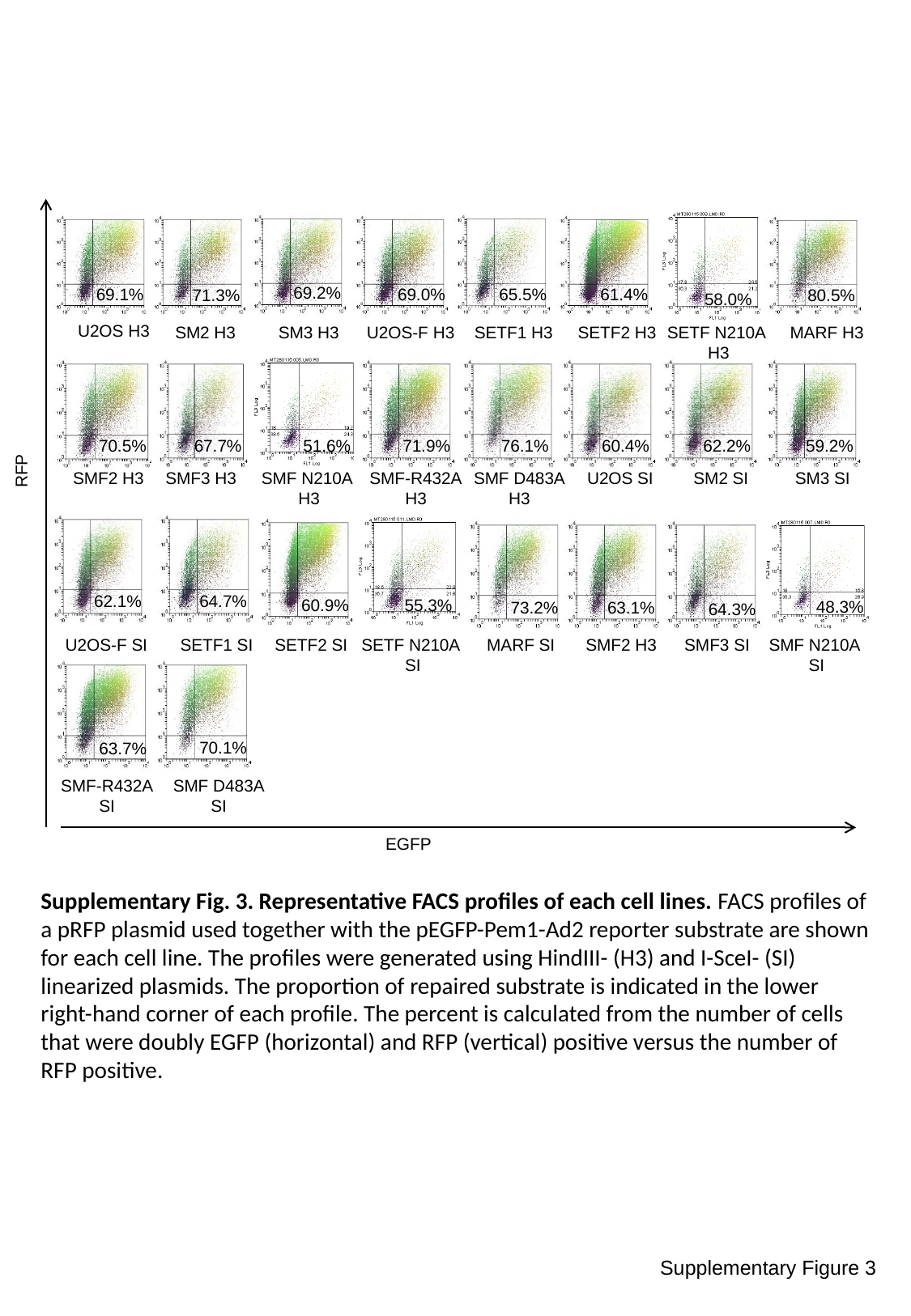

69.2%
69.1%
69.0%
65.5%
61.4%
71.3%
80.5%
58.0%
U2OS H3
SM2 H3
SM3 H3
U2OS-F H3
SETF1 H3
SETF2 H3
SETF N210A
H3
MARF H3
51.6%
67.7%
71.9%
76.1%
60.4%
62.2%
59.2%
70.5%
RFP
SMF2 H3
SMF3 H3
SMF N210A
H3
SMF-R432A
H3
SMF D483A
H3
U2OS SI
SM2 SI
SM3 SI
62.1%
64.7%
55.3%
60.9%
48.3%
73.2%
63.1%
64.3%
U2OS-F SI
SETF1 SI
SETF2 SI
SETF N210A
SI
MARF SI
SMF2 H3
SMF3 SI
SMF N210A
SI
70.1%
63.7%
SMF-R432A
SI
SMF D483A
SI
EGFP
Supplementary Fig. 3. Representative FACS profiles of each cell lines. FACS profiles of a pRFP plasmid used together with the pEGFP-Pem1-Ad2 reporter substrate are shown for each cell line. The profiles were generated using HindIII- (H3) and I-SceI- (SI) linearized plasmids. The proportion of repaired substrate is indicated in the lower right-hand corner of each profile. The percent is calculated from the number of cells that were doubly EGFP (horizontal) and RFP (vertical) positive versus the number of RFP positive.
Supplementary Figure 3
